# *Lespesia melloi* sp. nov. (Diptera: Tachinidae) from Brazil, a parasitoid of *Xanthopastis timais* (Lepidoptera: Noctuidae)

**DOI:** 10.1093/jis/14.1.121

**Published:** 2014-09-01

**Authors:** Helcio R. Gil-Santana, Silvio Shigueo Nihei, Enio Nunez

**Affiliations:** 1 Laboratorio de Diptera, Instituto Oswaldo Cruz. Avenida Brasil 4365, Manguinhos, 21045-900, Rio de Janeiro, RJ, Brasil; 2 Departamento de Zoologia, Instituto de Biociencias, Universidade de Sao Paulo, Rua do Matao, Travessa 14, n.101, 05508-090, Sao Paulo, SP, Brasil; 3 Centro Universitario Geraldo di Biase, Rodovia Benjamin Ielpo, Km11, 27101-090, Barra do Pirai, RJ, Brasil

**Keywords:** Amaryllis, Amaryllidaceae, taxonomy

## Abstract

A new species of the New World genus *Lespesia* , *Lespesia melloi***sp. nov.** (Diptera: Tachinidae), is described from southeastern Brazil. The species is reported here as a parasitoid of *Xanthopastis timais* (Cramer, 1782) (Lepidoptera: Noctuidae). The caterpillars of this noctuid feed on leaves and bulbs of amaryllis (Amaryllidaceae) in Brazil.

## Introduction

Flies of the family Tachinidae are known as endoparasitoids of other arthropods during their larval stage. Their hosts are predominantly other insects, including species belonging to eight orders, most of which are the lepidopteran larvae ( Wood and Zumbado 2010 ). Given this behavior, many tachinid species have great potential as biological control agents for forests and crop pests ( Stireman et al. 2006 , Nihei and Pavarini 2011 ).

In this article, we describe a new species of the New World tachinid genus *Lespesia* Robi-neau-Desvoidy, 1863 (Diptera: Tachinidae) from southeastern Brazil and report it as a parasitoid of *Xanthopastis timais* (Cramer, 1782) (Lepidoptera: Noctuidae).

Ranging from Canada to Chile, *Lespesia* is among the largest and most important genera of the New World Tachinidae ( Sabrosky 1980 ). The genus comprises 66 species, of which 37 occur in the Neotropical Region ( Guimarães 1983 , Toma 2010 ). Its species have been recorded parasitizing caterpillars from several families, particularly Noctuidae, Notodontidae, Saturniidae, and Sphingidae ( Guimarães 1977 , Wood and Zumbado 2010 ).

In South America, the species of *Lespesia* were revised by Guimarães (1983 , Brazilian spp.) and Toma (2010 , Venezuelan spp.). Scarce records of hosts for South American *Lespesia* spp. exist. The Argentinean *Pro-typophaemyia townsendi*Blanchard, 1963 (Exoristinae: Winthemiini) was reported as parasitoid of *Xanthopastis timais* (Cramer, 1782) (Lepidoptera: Noctuidae) in Santa Fé, Province of Corrientes, Argentina ( Blanchard 1963 , Guimarães 1977 ).

Caterpillars of *X. timais* preferentially attack plant species belonging to Amaryllidaceae ( Costa Lima 1950 ). This noctuid moth has a wide geographic range, from the southeastern United States, West Indies, throughout the Neotropical lowlands, and southward into northern Argentina ( Heppner 2000 ).

The amarillidaceans are commonly known as amaryllis ( *Amaryllis* spp.), and this plant species is sought after and cultivated by the public for its attractive appearance. Amaryllis, however, rarely grows in Brazil because of the serious and intense attacks from *X. timais* . The caterpillars invariably feed on leaves and bulbs, devouring those parts quickly and voraciously ( Monte 1932 , Figueiredo Jr. and Pereira 1944 ). Several authors have reported the economic damages of *X. timais* to lilies, particularly amarilidaceans ( Heppner 2000 ).

## Materials and Methods

**Fig. 1–2.**
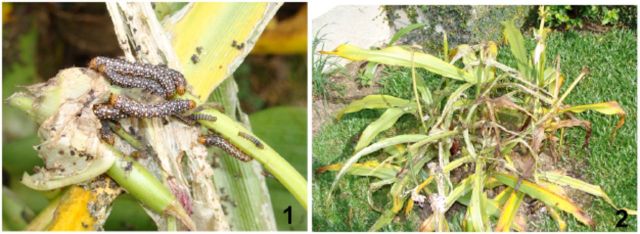
(1) Caterpillars of *Xanthopastis timais* feeding on *Amaryllis* sp.; (2) general view of *Amaryllis* sp. attacked by caterpillars of *X. timais* .

A population of caterpillars was found feeding on *Amaryllis* spp. (Amaryllidaceae) between May and June 2002 in the municipality of Nova Friburgo (22º 17´ S, 42º 29´ W; 1,010 m a.s.l), in the state of Rio de Janeiro, Brazil. Caterpillars were identified as *X. timais* ( Fig. 1 ). The plants had been intensively damaged by the caterpillars ( Fig. 2 ). Nineteen last-stage caterpillars were captured and separated into plastic containers with screened covers for subsequent pupation. Studied material was deposited at the Museu de Zoologia, Universidade de São Paulo, Brazil (MZSP) and the Entomological Collection, Museu Nacional da Universidade Federal do Rio de Janeiro, Rio de Janeiro, Brazil (MNRJ). The morphological terminology follows Cumming and Wood(2009 ) and Wood and Zumbado (2010 ).

**Fig. 3.**
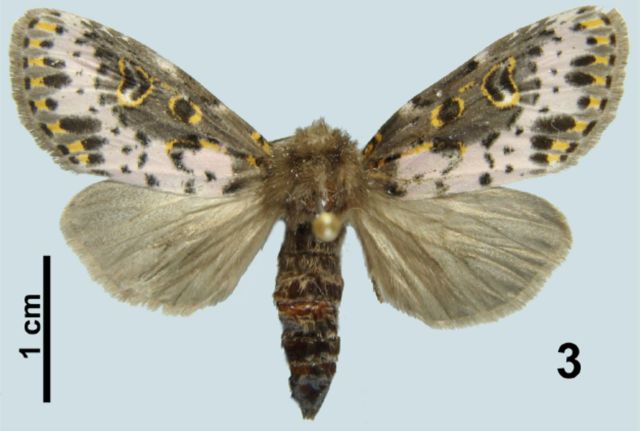
Adult of *Xanthopastis timais* .

**Fig. 4–8.**
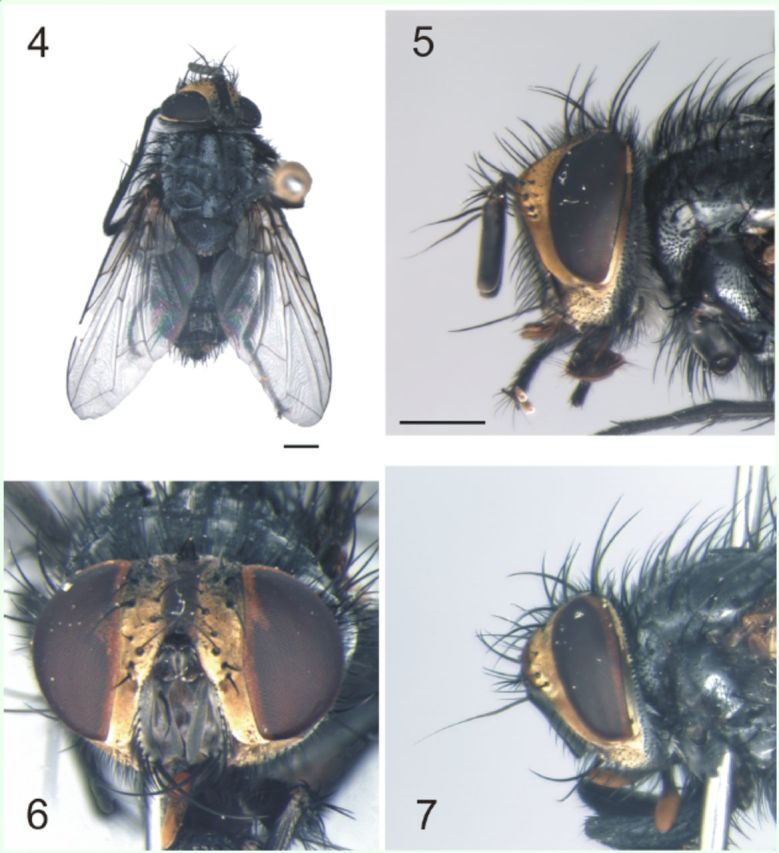
*Lespesia melloi* **sp. nov.** (4) male, dorsal habitus; (5) same, head, lateral view; (6) female, head, frontal view; (7) same, lateral view. (Scale bars: 1.0 mm).

Figures 1‒3 were made using a Sony Cyber-shot DSC H10 ( www.sony.com ); figures 4‒7 with a stereomicroscope Leica MZ16 with a digital camera ( www.leica-microsystems.com ), and the images were then combined by the software Automontage (Syncroscopy, www.syncroscopy.com ); and photograph 13 with a Leica microscope MC E with a digital câmera. Line drawings were made with a stereomicroscope Leica or Motic K700 with a camera lucida ( www.motic.com ).

### Nomenclature

This paper and the nomenclature it contains have been registered with ZooBank ( www.zoobank.org ). The LSID number is:

urn:lsid:zoobank.org :pub:E71DE41C-4C8A-4F90-8A54-1F2196ADBF20

## Results

During the period of 2–5 June 2002, only three caterpillars and two pupae were found parasitized among the 19 last instars of *X. timais* , and 11 tachinid larvae emerged. Other caterpillars were healthy and afterward emerged as adult moths ( Fig. 3 ). Two tachinid larvae were killed and preserved in 70% alcohol solution; the other larvae pupated a few hours after leaving the hosts. Adult flies emerged between 29 June and 1 July 2002.

The adults were examined and compared with species keys and revisions ( Sabrosky 1980 , Guimarães 1983 , Toma 2010 ). The material was compared with representatives of several species of *Lespesia* deposited at the MZSP. Based on that, we determined it as a new species of *Lespesia* Robineau-Desvoidy, 1863. This new species is described here. For a diagnosis of the genus *Lespesia* , please refer to Toma (2010 ), which provided a detailed diagnosis.

### *Lespesia melloi* sp. nov.

( Fig. 4 ‒11)

**Male.** Body length 6.9‒8.0 mm. Ratio frons width/ head width at vertex level 0.34‒0.35.

*Coloration* ( Fig. 4 ‒5): Fronto-orbital plate and parafacial deeply golden. Occiput black with white pruinosity; most occipital setulae white. Frons brownish and broad. Scape dark brown and without pruinosity; first flagellomere dark brown with white pruinosity; arista brown. Palpus yellow, covered by dark setulae; proboscis dark brown. Gena with golden pruinosity. Thorax dark-brown colored, nearly black, with four distinct stripes and white pruinosity. Scutellum dark brown, nearly black, but brown on apex, with white pruinosity. Wing hyaline; calypters white; haltere yellow. Legs dark-brown, nearly black, with white pruinosity on fore coxa ventrally. Abdomen blackish, T1+2 with white pruinosity, more conspicuous on ventral side, T3 with white pruinosity, T5 with strong golden pruinosity and brown apex. *Head* ( Fig. 5 ‒7): Eyes slightly haired. Seven frontal setae, three of which inserted below the level of antennal insertion. Frons width about 1/7 of the head width. Two reclinate fronto-orbital setae at the upper portion. Fronto-orbital plate densely covered by delicate fine setulae mostly on upper half. Ocellar setae proclinate and as strong as reclinate fronto-orbital setae. Several fine and long postocellar setulae, no developed seta. Inner vertical setae strong, slightly convergent and reclinate; the outer vertical setae weak, divergent, less than half the length of the inner. First flagellomere elongated, about 5× its width, almost reaching the lower facial margin. Arista bare, about the same length as antenna, and narrowing abruptly on the upper half. Parafacial bare. Facial ridge with setulae from vibrissa almost until the level of insertion of arista. Vibrissae long and cruciate.

**Fig. 8–11.**
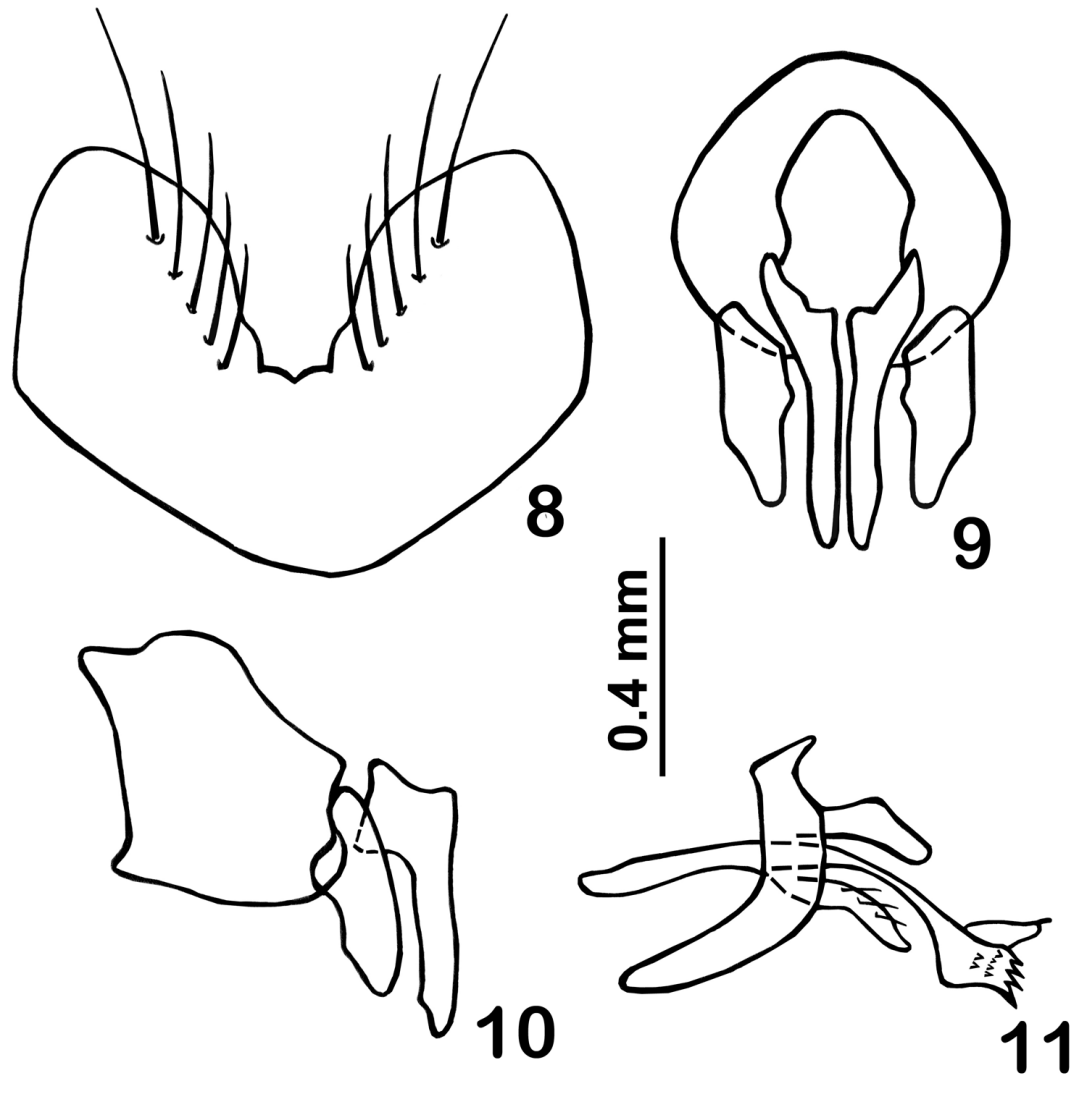
*Lespesia melloi* **sp. nov.** , male terminalia. (8) fifth sternite; (9) epandrium, cerci and surstyli, in posterior view; (10) same, in lateral view; (11) hypandrium, postgonite, pregonite and distiphallus, in lateral view.

Gena about 1/6 of the eye height. *Thorax:* Acrostichals 3+3; dorsocentrals 3+4; supra-alars 1+3, the first postsutural one strong (stronger than the first post-dorsocentral seta); intra-alars 2+3, the first presutural seta beside the postpronotal lobe and the second presutural seta very close to the suture. One developed seta between the first presutural intra-alar and first presutural supra-alar. Intra-postalar seta present. Postpronotals 4. Notopleurals 2. Prosternum setulose. Proepisternum bare. Katepisternals 2+2, the lower ones weaker. Posterior spiracle rounded and covered by a flap-shaped tuft of setulae. Scutellum with one pair of basal and subapical setae longer and stronger than lateral and discal pair of setae, and one apical cruciate pair, the latter longer than discal but shorter than lateral pair. *Wings:* Cell R4+5 open, the distance between apex of R4+5 and M as long as r-m crossvein. Base of R4+5 setulose dorsally and ventrally; remaining veins bare. *Legs:* Tarsal claws as long as or slightly longer than last tarsomere. Fore femur with posterodorsal and posteroventral rows of setae, the posterior face well-developed setae forming an irregular row; fore tibia with anterodorsal row of setae on the mid third, two strong posterior setae on the mid third; tarsal pulvilli as long as the claws. Mid femur with weak anteroventral row of setae and strong posteroventral row, about 3-4 strong median setae on anterior face, and three preapical oblique posterodorsal setae; mid tibia with one strong median anterodorsal seta, one submedian ventral seta, and two posterior setae on the mid third. Hind femur with anterodorsal and anteroventral rows of setae, a posteroventral row of setae, with long setae on the basal half, and two preapical oblique posterodorsal setae; hind tibia with an anterodorsal row of setae of subequal size, but one long submedian seta, 3-4 anteroventral setae, the basal ones weaker, and two posterodorsal setae on the mid third. *Abdomen:* T1+2 excavated until the posterior margin, with a pair of lateral marginal setae; T3 with a pair of median marginal setae and two pairs of lateral marginal setae; T4 with a row of marginal setae reaching the ventral margin; T5 with a row of marginal setae, with irregular discal setae, and covered by setulae more separated from each other than on previous tergites. *Male terminalia* ( Fig. 8 ‒11): Sternite 5 as in Fig. 8 . Cerci slightly broadened at base, tapering toward the apex in posterior view ( Fig. 9 ), the tip elongated and narrow and slightly curved inwards, ending beyond the apex of surstyli. Surstyli, in lateral view ( Fig. 10 ), broad and the apex round but the middle narrow; in posterior view ( Fig. 9 ) with a median projection on the inner surface. Pregonite ( Fig. 11 ) narrowing towards the apex and covered by few cilia; postgonite connected to hypandrium. Epiphallus slightly broadened at base, but uniformly narrowed until the apex. Distiphallus composed by a broad dorsal sclerite, with an elongated and narrow membrane ( Fig. 11 ).

**Female.** Body length 6.9‒7.0 mm. Ratio frons/ head width at the vertex level: 0.36‒0.38. Differs from male as follows: two proclinate fronto-orbital setae; five frontal setae, three of which are below the insertion of antenna; aristas slightly broader; palpus orangish and conspicuously broadened, with dark setulae restricted to base; tarsal claws shorter than last tarsomere.

**Fig. 12.**
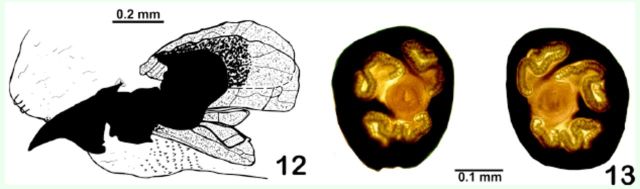
Third instars of *Lespesia melloi***sp. nov.** (12) cephalopharyngeal skeleton; (13) posterior spiracles.

**Third instar** ( Fig. 12 ‒13). Length 9.0 mm. General color whitish gray; cephalopharyngeal skeleton ( Fig. 12 ) characterized by a conspicuous mandible and hypopharynx; tentorial phragma heavily sclerotized; dorsal and ventral cornus expanded, less sclerotized. Posterior spiracles ( Fig. 13 ) heavily sclerotized with four very sinuous openings, two of which are shorter and adjacent to the others which are separated by thin prolongations of the wall of the spiracle.

**Type material examined.** Holotype male, BRAZIL, Rio de Janeiro, Nova Friburgo (22° 17'S-42°29'W), 29.vi-02.vii.2002, H. R. Gil-Santana *leg.(MZSP).***Paratypes:** same data, 2 males and 3 females (MZSP), 1 male and 2 females (MNRJ), with same data, also two third instars.

**Etymology.** The new species is named in homage to Dr. Rubens Pinto de Mello for his great contributions to the study and teaching of entomology in Brazil, particularly those on Diptera.

**Remarks.** *Lespesia melloi* **sp. nov.** resembles *L. lata* (Wiedemann, 1830) and *L. lanei*Guimarães, 1983 by its fronto-orbital plate and parafacial strongly golden pruinose, but differs from the former by the bare eyes and two lateral scutellar setae (eyes densely setulose and three lateral scutellars in *L. lata).* Nevertheless, the new species is morphologically very similar to *L. lanei,* and it runs to couplet 5 of the key provided by Guimarães (1982: 12) along with *L. lanei.* And when compared with the type-series of *L. lanei* deposited at the MZSP, *L. melloi***sp. nov.** can be distinguished by the following features:

(1) Frontal vitta black (brownish in *L. lanei).*

(2) Guimarães (1983 , 19) described *L. lanei* with “Parafrontalia, and parafacialia, yellow to golden pollinose,” but in the key to males provided on p. 12, he described them as having “parafrontalia, parafacialia golden yellow tomentose” (couplet 5). We examined the holotype male of *L. lanei* and confirmed it only has a pale golden yellow tomentum, which is not as strong as the golden egg yellow tomentosity of *L. melloi.*

(3) Guimarães (1983 ) described the abdominal tergite 1+2 with “a small but distinct pair of median marginal,” but in the key to males, he characterized it with “two pairs of small me dian marginal.” Following our examination, the holotype male of *L. lanei* has a small yet distinguishable pair of median marginals with a very weak pair between this pair. The new species has no developed or distinguishable median marginals.

(4) The cerci and surstyli of *L. lanei* ( Guimarães, 1983 , Fig. 12 ) are clearly differ ent from those of *L. melloi* . In *L. lanei* , the base of cerci is narrow in posterior view, and the apex rounded in lateral view; in *L. melloi* , the base is wider and the apex rather pointed. The surstyli of *L. lanei* are more rounded either in posterior or lateral view; in *L. melloi* they are trapezoid and pointed.

## References

[R1] Blanchard E. E. 1963 . Dipteros parásitos de Noctuidae argentinos . *Rev. Investig. Agríc.* 17 : 129 – 254 .

[R2] Cumming J. M. Wood D. M. . 2009 . Adult morphology and terminology, Ch. 2 . *In* B. V. Brown et al. (eds.) . *Manual of Central American Diptera* . Vol. 1. NRC Research Press, Ottawa.

[R3] Costa Lima A. 1950 *. Insetos do Brasil* . Rio de Janeiro. 6° Tomo. Lepidópteros, 2ª Parte.

[R4] Figueiredo E. R. Jr Pereira H. F. . 1944 . Notas sôbre “ *Xantophastis timais* ” (Cram.) (Lep. Noct.), praga das amarilidáceas . *Arquivos Institut. Biol.* 15 : 289 – 298 .

[R5] Guimarães J. H. 1977 . Host-parasite and parasite-host catalogue of South American Tachinidae (Diptera) . *Arquivos Zool* . 29 : 1 – 131 .

[R6] Guimarães J. H. 1983 . Taxonomy of Brazilian flies of the genus *Lespesia* Robineau-Desvoidy (Diptera, Tachinidae) . *Pap. Avulsos Zool* . 35 : 11 – 30 .

[R7] Heppner J. B. 2000 . Spanish moth, *Xanthopastis timais* (Lepidoptera: Noctuidae): a pest of amaryllis and other lillies . *Fla. Dep. Agric. Consumer Serv. Entomol. Circ* . 401 .

[R8] Monte O. 1932 Notas biológicas sobre o lepidóptero noctuideo *Xantophastis timais* , Cram. *O Campo* 3 : 41 – 42 .

[R9] Nihei S. S. Pavarini R. . 2011 . Taxonomic redescription and biological notes on *Diaugia angusta* (Diptera, Tachinidae): parasitoid of the palm boring weevil *Metamasius ensirostris* and *M. hemipterus* (Coleoptera, Dryophthoridae). *Zookeys* 84 : 23 – 38 . 10.3897/zookeys.84.756 PMC3088068 21594164

[R10] Sabrosky C. W. 1980 . A revised key to the Nearctic species of *Lespesia* (Diptera: Tachinidae) . *Ann. Entomol. Soc. Am* . 73 : 63 – 73 .

[R11] Stireman J. O. O'fHari D. M. . 2006 . Tachinidae: evolution, behavior, and ecology . *Annu. Rev. Entomol* . 51 : 525 – 555 . 16332222 10.1146/annurev.ento.51.110104.151133

[R12] Toma R. 2010 . Contribuicao ao conhecimento das especies venezuelanas de *Lespesia* Robineau-Desvoidy (Diptera, Tachinidae, Exoristinae), com descricao de novas especies . * Rev. Brasil. Entomol* . 54 : 165 – 172 .

[R13] Wood D. M., Zumbado M. A. 2010 . Tachinidae (Tachinid Flies, Parasitic Flies), Ch. 113. . *In B. V. Brown et al. (eds.). Manual of Central American Diptera, NRC Research Press* . 2 .

